# Effectiveness of a multiple health-behaviour-change intervention in increasing adherence to the Mediterranean Diet in adults (EIRA study): a randomized controlled hybrid trial

**DOI:** 10.1186/s12889-022-14590-y

**Published:** 2022-11-19

**Authors:** Jose I. Recio-Rodriguez, Luis Garcia-Ortiz, Irene A. Garcia-Yu, Cristina Lugones-Sanchez, Edurne Zabaleta-del Olmo, Bonaventura Bolibar, Marc Casajuana-Closas, Tomas Lopez-Jimenez, Joan Llobera, Rafel Ramos, Haizea Pombo, Emma Motrico, Montserrat Gil-Girbau, Fatima Lopez-Mendez, Francisco Represas-Carrera, Jose A. Maderuelo-Fernandez

**Affiliations:** 1 Unidad de Investigación de Atención Primaria de Salamanca (APISAL), Instituto de Investigación Biomédica de Salamanca (IBSAL), Red de Investigación en Cronicidad, Atención Primaria Y Promoción de La Salud (RICAPPS) (RD21/0016), Facultad de Enfermería Y Fisioterapia (Universidad de Salamanca), Salamanca, Spain; 2 Unidad de Investigación de Atención Primaria de Salamanca (APISAL), Instituto de Investigación Biomédica de Salamanca (IBSAL), Gerencia de Atención Primaria de Salamanca, Departamento de Ciencias Biomédicas Y del Diagnóstico (Universidad de Salamanca), Gerencia Regional de Salud de Castilla Y León (SACyL), 37007 Salamanca, Spain; 3 Unidad de Investigación de Atención Primaria de Salamanca (APISAL), Instituto de Investigación Biomédica de Salamanca (IBSAL), Salamanca, Spain; 4 Unidad de Investigación de Atención Primaria de Salamanca (APISAL), Instituto de Investigación Biomédica de Salamanca (IBSAL), Gerencia de Atención Primaria de Salamanca, Gerencia Regional de Salud de Castilla Y León (SACyL), Salamanca, Spain; 5 Nursing Department, Nursing Faculty (Universitat de Girona), Fundació Institut Universitari Per a La Recerca a L’Atenció Primària de Salut Jordi Gol I Gurina (IDIAPJGol), Gerència Territorial de Barcelona (Institut Català de La Salut), UniversitatAutònoma de Barcelona, Barcelona, Spain; 6 Fundació Institut Universitari Per a La Recerca a L’Atenció Primària de Salut Jordi Gol I Gurina (IDIAPJGol), Universitat Autònoma de Barcelona, Barcelona, Spain; 7 Fundació Institut Universitari Per a La Recerca a L’Atenció Primària de Salut Jordi Gol I Gurina (IDIAPJGol), Barcelona, Spain; 8 Unitat de Recerca, Atenció Primària de Mallorca. Servei de Salut de Les Illes Balears. IdISBa, Palma, Spain; 9 Fundació Institut Universitari Per a La Recerca a L’Atenció Primària de Salut Jordi Gol I Gurina (IDIAPJGol), Group of Research in Vascular Health, Unitat de Suport a La Recerca de Girona, Girona Biomedical Research Institute (IdibGi), Girona, Spain; 10 Bizkaia, Deputy Directorate of Healthcare Assistance, Ezkerraldea-Enkarterri-Cruces Integrated Health Organisation-Biocruces Bizkaia Health Research Institute Innovation Unit, Osakidetza-Servicio Vasco de Salud, Araba, Spain; 11 Universidad Loyola Andalucía, Seville, Spain; 12 Research and Development Unit, Institut de Recerca Sant Joan de Déu, Sant Boi de Llobregat, Spain; 13 Instituto de Investigación Sanitaria Aragón, Saragossa, Spain; 14 Atención Primaria, Área Sanitaria de Vigo, Servicio Gallego de Salud (SERGAS), Grupo I-Saúde (Instituto de Investigación Sanitaria Galicia Sur), Galicia, Spain

**Keywords:** Complex interventions, Cost-effectiveness analysis, Health behavior, Health promotion, Hybrid trial, Implementation research, Mediterranean diet, Physical activity, Primary health care, Smoking

## Abstract

**Background:**

The present study describes the effectiveness of a complex intervention that addresses multiple lifestyles to promote healthy behaviours in increasing adherence to the Mediterranean diet (MD).

**Methods:**

Cluster-randomised, hybrid clinical trial controlled with two parallel groups. The study was carried out in 26 primary Spanish healthcare centres. People aged 45–75 years who presented at least two of the following criteria were included: smoker, low adherence to the MD or insufficient level of physical activity. The intervention group (IG) had three different levels of action: individual, group, and community, with the aim of acting on the behaviours related to smoking, diet and physical activity at the same time. The individual intervention included personalised recommendations and agreements on the objectives to attain. Group sessions were adapted to the context of each healthcare centre. The community intervention was focused on the social prescription of resources and activities performed in the environment of the community of each healthcare centre. Control group (CG) received brief advice given in the usual visits to the doctor’s office. The primary outcome was the change, after 12 months, in the number of participants in each group with good adherence to the MD pattern. Secondary outcomes included the change in the total score of the MD adherence score (MEDAS) and the change in some cardiovascular risk factors.

**Results:**

Three thousand sixty-two participants were included (IG = 1,481, CG = 1,581). Low adherence to the MD was present in 1,384 (93.5%) participants, of whom 1,233 initiated the intervention and conducted at least one individual visit with a healthcare professional. A greater increase (13.7%; 95% CI, 9.9–17.5; *p* < 0.001) was obtained by IG in the number of participants who reached 9 points or more (good adherence) in the MEDAS at the final visit. Moreover, the effect attributable to the intervention obtained a greater increase (0.50 points; 95% CI, 0.35 to 0.66; *p* < 0.001) in IG.

**Conclusions:**

A complex intervention modelled and carried out by primary healthcare professionals, within a real clinical healthcare context, achieved a global increase in the adherence to the MD compared to the brief advice.

**Trial registration:**

ClinicalTrials.gov Identifier: NCT03136211. Retrospectively registered on 02/05/2017 https://clinicaltrials.gov/ct2/show/NCT03136211

## Background

One of the most studied dietary patterns, and with the most accumulated scientific evidence regarding its health benefits, is the Mediterranean Diet (MD). It is the traditional dietary pattern of the countries bordering the Mediterranean Sea, with variations depending on the area due to culinary preferences and sociocultural and religious factors. These are identified as the main characteristics of this diet [1]: 1) High consumption of fruits and vegetables, legumes, nuts and whole grains; 2) Consumption of local, fresh and seasonal food; 3) The use of extra-virgin olive oil as the main source of lipids; 4) Moderate consumption of eggs, fish, shellfish and white meat 5) Frequent but moderate consumption of wine, mainly red, with meals; 6) Low consumption of sweets, red meat, processed meat and dairy products. The Mediterranean Diet has been associated, among others, with a lower risk of suffering from cardiovascular diseases (2,3), with a reduction of total mortality [2], as well as with the reduction of the risk of metabolic syndrome and its components, such as obesity, hypertension and hyperlipidemia [3]. Adherence to the traditional pattern of this diet in southern Europe is progressively declining [4]. This decrease in adherence seems to be related to the increase in the prevalence of obesity, although it is unknown if it is a causal relationship. To this fact must be added the change in several aspects related to food, such as the availability and variety of products, as well as the change in dietary behaviors, such as the intake of soft drinks and fast food [5].

The increase of adherence to the MD has also been a target of multicomponent and multibehavioural interventions. Alonso-Dominguez et al. [6] achieved an increase of 2.2 points in the Mediterranean Diet Adherence Score (MEDAS) after an intervention in people with type 2 diabetes mellitus, which combined heart-healthy walks, the use of a smartphone application and a food workshop, compared to the control group, who only received counseling. However, the results of interventions that only included the use of new technologies have shown a lesser impact [7]. The systematic review by Maderuelo et al. [8] collected studies that included interventions to improve adherence to the MD. In one of these studies (Zazpe et al. [9], PREDIMED study), the two intervention groups, who followed a programme based on motivational strategy, submission of written material and the intake of supplements (olive oil or nuts), increased their MEDAS score by 1.86 and 2.26 points, respectively, whereas the control group, who followed a low-fat diet, only increased this score by 0.46 points. On the other hand, Logan et al. [10] found no differences in adherence to the MD after the intervention among the three study groups (nutritional advice, behavioural changes and control group).

In all the studies mentioned above, adherence to the MD was estimated with a diet quality index, the MEDAS. This is a valid instrument for rapid estimation of adherence to the MD and may be useful in clinical practice. The MEDAS was validated in a large Spanish population and correlated significantly with the corresponding food-frequency questionnaire (FFQ) PREDIMED score (*r* = 0.52; intraclass correlation coefficient = 0.51) and in the anticipated directions with the dietary intakes reported on the FFQ [11].

The EIRA study has as a novelty the inclusion of a complex intervention to achieve the modification of lifestyles. Complex interventions promote the participation of professionals and citizens throughout the design, execution and analysis of research, which, in turn, increases and improves the transference of results to clinical practice [12]. The EIRA study [13], through a hybrid trial, evaluates, as the main objective, the cost-effectiveness and implementation of a complex intervention that addresses multiple lifestyles (diet, physical activity and smoking) to promote healthy behaviours in a large sample of patients between 45 and 75 years of age attended in primary healthcare. The full trial results were published separately [14, 15]. This manuscript reports the effectiveness of this intervention to increase adherence to the MD.

## Methods

### Design

This is a cluster-randomised, type 2 hybrid clinical trial controlled with two parallel groups. The protocol of the clinical trial [13] was written and published in compliance with the Recommendations for Interventional Trials (SPIRIT) [16] and the Standards for Reporting Implementation Studies (StaRI) [17].

### Study setting

The study was carried out in primary healthcare centres of seven Autonomous Communities of Spain from January 2017 to December 2018. Healthcare in this scope is provided in healthcare centres, which are manned by multidisciplinary teams of physicians, nurses, pediatricians, midwives, social workers and dentists, who carry out activities of health care, health education, health promotion and prevention and activities for the community [18].

### Recruitment

The participants were recruited from the health centres through several methods: 1) at the time of the visit as part of usual care; 2) waiting room or admission desk of the healthcare centre; 3) through a person in charge of recruiting participants; 4) posters in the health centres; 5) phone calls to patients who met the selection criteria.

### Participants

The EIRA study includes the participation of 26 healthcare centres. The criteria for the selection of the centres were the following: 1) having access to the Internet; 2) having the possibility of carrying out community activities; 3) being located in areas without great cultural and linguistic diversity; and 4) having an active and highly committed professional team. The professionals of the selected healthcare centres participated voluntarily and signed a collaboration commitment to the study.

The study included people between 45 and 75 years of age who presented at least two of the following criteria related to their lifestyles at the time of the screening: 1) smoker; 2) low adherence to the MD (evaluated through two validated questions about the daily consumption of fruits and vegetables [19]); and 3) insufficient level of physical activity (evaluated through the Brief Physical Activity Assessment Tool [20]). The exclusion criteria were as follows: advanced serious illnesses, cognitive impairment, dependence in basic everyday activities, severe mental illness, being included in a long-term home health care program, being under treatment for cancer or in end-of-life care, or not planning to reside in the area during the time that the intervention lasted. The selected participants signed an informed consent before any examination or procedure of the study and conducted two evaluations, i.e., at baseline and at 12 months.

### Data collection and management

Recruitment, screening visit and intervention visits, including group sessions, were performed by the healthcare professionals of the participating healthcare centres. The evaluation visits (baseline and 12 months) were conducted by an external unit of healthcare professionals trained in each centre since these activities were not considered to be related to the usual care. An electronic data-collection notebook was designed. The study was managed by coordinators at the central, regional and local levels within each of the participating healthcare centres. Different communication methods, such as meetings and newsletters, were used among the coordinators and managers of the study.

### Primary and secondary outcomes of this manuscript

This hybrid trial has the following primary objectives: to evaluate the effectiveness and cost-effectiveness of a complex multiple risk intervention on reducing tobacco use, enhancing adherence to Mediterranean dietary pattern and increasing physical activity level in 12 months to baseline compared with usual care; to assess the effectiveness of an implementation strategy in terms of acceptability, adoption, appropriateness, feasibility, fidelity, implementation cost and penetration.

The primary outcome of this manuscript was the change, after 12 months, in the number of participants in each group with a score equal to or greater than 9 points on the MEDAS, which would indicate good adherence to the MD pattern. Secondary outcomes were the change in the global score of adherence to the MD, the change in the percentage of individuals who positively complied with each of the items of the questionnaire, and the change in weight, blood pressure and laboratory variables included in cardiovascular risk estimation calculators.

### Measurement variables and instrument

#### Adherence to the Mediterranean Diet

This questionnaire was developed and validated by the PREDIMED group [11], and includes 14 questions with two possible answers. Each item was scored with either zero or one point depending on whether the individual met the established recommendations regarding the MD. These recommendations are the following: 1) use of olive oil as the main fat for cooking; 2) daily consumption of at least 4 tablespoons of olive oil; 3) daily intake of at least 2 servings (1 serving = 200 g) of vegetables (at least one of them in a salad or raw); 4) daily consumption of 3 or more fruit pieces (including natural juice); 5) intake of less than one carbonated and/or sugary beverage per day; 6) consumption of less than one serving (100–150 g) of red meat, hamburgers, sausages or processed meat per day; 7) weekly consumption of at least 3 servings (1 serving = 150 g) of legumes; 8) consumption of at least 3 servings of fish or shellfish per week (1 piece or portion dish: 100—150 fish or 4–5 pieces or 200 g of seafood); 9) consumption of less than one serving (12 g) of cream, butter or margarine per day; 10) consumption of 7 or more glasses of wine in the whole previous week; 11) intake of nuts 3 or more times per week (1 serving = 30 g); 12) intake of industrial (non-home-made) pastries less than twice per week; 13) preference for the consumption of chicken, turkey or rabbit meat instead of beef, pork, hamburgers or sausages; and 14) consumption of “sofrito” (sauce made with tomato, garlic, onion and other vegetables to dress rice, pasta, meat and other dishes) 2 or more times per week. The final score range was 0 to 14 points. Although the MEDAS questionnaire does not have an established cut-off point to discern good adherence to the MD, the cut-off point of 9 points or more has frequently been used to indicate good adherence to this dietary pattern [6, 21].

#### Clinically relevant measures

Other variables were measured, including medication use obtained from medical notes, blood pressure, glucose, weight, height, and laboratory parameters (total serum cholesterol, LDL-cholesterol, HDL-cholesterol, triglycerides). Weight was measured twice at each visit using validated scales with an error of ± 0.1 kg. Blood pressure was measured 3 times in each arm using validated devices (OMRON M10-IT) following the measurement protocol of the European Society of Hypertension [22]. Laboratory tests were performed on fasting for 10–12 h and samples were sent to a central laboratory to be analyzed. The central laboratories all belonged to hospitals of the Spanish Health System and met all the required quality requirements. Cholesterol and triglyceride values were expressed in mg/dL and glycosylated hemoglobin in percentage. A detailed description of the way in which these variables were measured was published with the study protocol [13].

On the first visit, a researcher asked each individual questions to determine the stage of change for each lifestyle studied following the constructs of the Transtheoretical Model by Prochaska et al. [23] that classifies individuals into various stages of health behavior change: Precontemplation: No intention to take action within the next 6 months, Contemplation: Intends to take action within the next 6 months, Preparation: Intends to take action within the next 30 days and has taken some behavioural steps in this direction, Action: Changed overt behaviour for less than 6 months, Maintenance: Changed overt behaviour for more than 6 months, and Termination: No temptation to relapse and 100% confidence.

### Assignment of intervention

The assignment of the intervention was performed by healthcare centre and was computer-generated at a central location (IDIAP Jordi Gol, Barcelona, Spain). For each of the seven Autonomous Communities, half of their participating healthcare centres (*N* = 13) were randomised to the intervention group and the other half (*N* = 13) were randomised to the control group. Both the investigators conducting the assessments (baseline and 12 months) and the investigators conducting the statistical analyzes were blinded to the intervention. Due to the very characteristics of the intervention, both the participants and the researchers who carried out the interventions could not be blinded to the intervention.

### Intervention (only healthcare centres of the intervention group)

The intervention was grounded in the transtheoretical model of behaviour change [24] and was conducted by physicians and nurses of the healthcare centres within their routine of usual care, according to the conceptual framework of the “5 A’s”: Assess, Advise, Agree, Assist, and Arrange follow-up [25]. The intervention was based on the results of the previous phases of the study (pre-clinical, phase I and phase II) [8, 26–32]. It had three different action levels: individual, group and community. In all these levels, the aim was to act on the behaviours related to smoking, diet and physical activity at the same time, although, depending on the participant, actions were prioritised for one or more of these behaviours.

Individual intervention: the individual intervention level included 2–3 visits of 25–30 min each, with the possibility of conducting one more visit as a reinforcement of the intervention. Depending on the stage of change in which each participant was for each of the behaviours, either very brief (3 min) intervention (participants in pre-contemplation, contemplation and maintenance phase) or specific intervention (participants in preparation and action phase) was provided. The aim of the very brief intervention was to raise awareness of the need for a behavioural change and support the change or help to prevent possible relapses, whereas the specific intervention was aimed at establishing an agreed specific plan for the behavioural change. To guarantee an adequate and standardized measurement of the participants' stage of change in relation to their lifestyles, all the professionals undertook a 20-h online course on motivational interview [33]. Some studies suggest the effectiveness of the Short Message Service-SMS in health promotion interventions, especially in relation to the cessation of tobacco consumption and increasing physical activity, although certain gaps persist and further research on the subject is recommended [34, 35]. The intervention employed information and communication technologies (ICTs), such as websites for the participants, text messaging (in all the stages of change), smartphone applications (only in the preparation and action phases) [7] and other assistive devices like smartwatches and pedometers. The selection of the ICTs was carried out by previous pilot study and the effectiveness of these in previous studies [7]. Its relationship with the theory of change is described in Table 1.

**Table 1:** Characteristics of nutritional component of the intervention according to the dietary habits stage of change

	Individual	Group	Community
Precontemplation	Very brief (3 min) intervention + SMS^a^		
Contemplation			
Preparation	Brief (12–15 min) Intervention + information and communication technologies (smartphone applications + SMS^a^)	Health education workshops	Social prescribing
Action			
Maintenance	Very brief (3 min) intervention + SMS^a^		
Termination			

^a^*SMS* Short Message Service

Group intervention: Two health education workshops focused on healthy diet and physical activity were carried out. These workshops were carried out after the individual intervention and were directed by professionals of the involved centres. They had a duration of 90–120 min and were aimed at strengthening the recommendations given in the individual sessions and providing guidelines to facilitate the practice of physical activity and the adoption of healthy eating behaviours. The group sessions were adapted to the context of each healthcare centre of the participating intervention group. Some examples of workshops were: physical exercise sessions, cooking workshops or preparing seasonal menus.

Community intervention: This intervention was focused on the social prescription [36] of resources and activities performed in the environment of the community of each healthcare centre. Social prescription, also sometimes known as community referral, is a means of enabling health professionals to refer people to a range of local, non-clinical services. The referrals generally, but not exclusively, come from professionals working in primary care settings, for example, general practitioners or practice nurses. In the EIRA study, previously, each centre identified the available resources, as well as the activities with their schedule, duration and frequency. Depending on the possibilities of each participant and other conditioning factors like the work environment and time availability, the activities that best suited each participant were selected. Some examples of these activities were: cooking courses, heart-healthy walks, sport activities, dancing courses and activities, and green physical activity programmes. Its relationship with the theory of change is described in Table 1.

Nutritional component of the intervention: The nutritional intervention was carried out by a person included as staff of each Primary Care Team participating in the study. A specific training in dietary advice and on the Mediterranean diet was provided to the professionals who were in charge of carrying out this intervention in each center. The training lasted 8 h and was imparted by a dietician-nutritionist. Within the individual intervention, the specific section about MD included the following contents: 1) explanation of the concept of MD; 2) reasons for adopting a MD; 3) personalised recommendations about the changes that must be introduced and agreement on the objectives to attain; 4) delivery of informative material summarizing the intervention (leaflets); 5) the possibility of carrying out group workshops; 6) the possibility of conducting community activities; 7) the possibility of joining the SMS messaging programme; 8) the possibility of using ICTs (websites, smartphone applications, etc.). Within the group intervention, the specific section about the MD included health education workshops, cooking workshops and preparing seasonal menus. Community intervention was based on social prescription that included the activities identified in each intervention center. Some examples of these activities were cooking courses or nutrition education sessions to the community. Table 1 includes information on the type of intervention based on the stage of change in nutritional habits.

The specific intervention to address smoking followed the guidelines of motivational interview and included the establishment of a D-day to quit smoking and the submission of written material with follow-up visits at 15 days and 1 month after the D-day. On the other hand, the specific intervention to address physical activity was based on consensus with the participant on a personalised physical activity plan that preferably included community resources or programmes in the healthcare centre itself.

### Usual care (healthcare centres of the control group)

The professionals of the control group healthcare centres were requested to integrate the lifestyle recommendations of the Programme of Preventive Activities and Health Promotion [37] into their usual practice. This programme includes protocols about lifestyle recommendations (diet recommendations focusing on the adoption of the MD, physical activity and smoking cessation) and a set of preventive activities for specific groups of patients (i.e., age, sex and risk) based on brief advice for the prevention of cardiovascular diseases, mental diseases and cancer, as well as for the follow-up of general and specific vaccination campaigns. This intervention in the control group differs from the EIRA intervention, since it is a brief, non-intensive and unique intervention.

### Ethics approval

The template informed consent forms were reviewed and approved by the Research Ethics Committee of the IDIAP Jordi Gol (approval number P16/025). The study complied with all applicable laws on the protection of personal data. All data collection forms were identified by a coded identification number only to maintain participant confidentiality.

### Sample size

A sample size of 3640 participants (1820 for each group), allowing for 30% loss to follow-up, was estimated to have 80% power (at 5% significance level, two-tailed and with an intracluster correlation of 0.01), to detect an absolute difference in a positive change in one or more of the three behaviours of 8% between groups (EIRA intervention and usual care).

Statistical power was estimated for the main outcome variable of this manuscript (percentage of individuals with good adherence to the MD at the end of the study). Accepting an alpha risk of 0.05 and a power of 80% in a bilateral test, a sample of 1481 subjects in the first group (EIRA intervention) and 1581 in the control group was enough to recognize as statistically significant the difference from 42% in the first group to 34% in the second group (statistical power 100%).

### Statistical analyses

The statistical analyses were conducted by intention-to-treat. The normality of the variables was assessed using the Kolmogorov–Smirnov test. To address potential biases due to incomplete follow-up and non-response in the surveys, multiple imputation by chained equations (mice function in R software) with 50 imputed datasets was applied to outcomes and covariates [38, 39]. Estimates from each imputed dataset were combined following the rules outlined by Rubin [40]. The categorical variables are expressed as *n* and percentages, whereas the quantitative variables are expressed as mean and standard deviation. The mean differences between the study groups were evaluated with Student’s t-test, while the intragroup differences were assessed through Student’s t-test for paired data. The relationship between two categorical variables was analysed using the chi-squared test. Analysis of covariance (ANCOVA), with age, sex, center and dietary habits stage of change as covariates, was used to compare the changes between the two groups. For secondary outcomes, changes in physical activity and smoking cessation as covariates were additionally included in the analysis model. For the bilateral contrast of hypotheses, an alpha risk of 0.05 was set as the limit of statistical significance. The data were analysed using the statistical software SPSS for Windows version 25.0. (IBM Corp, Armonk, NY, USA).

## Results

### Participants and characteristics in the baseline evaluation

**Fig. 1:**
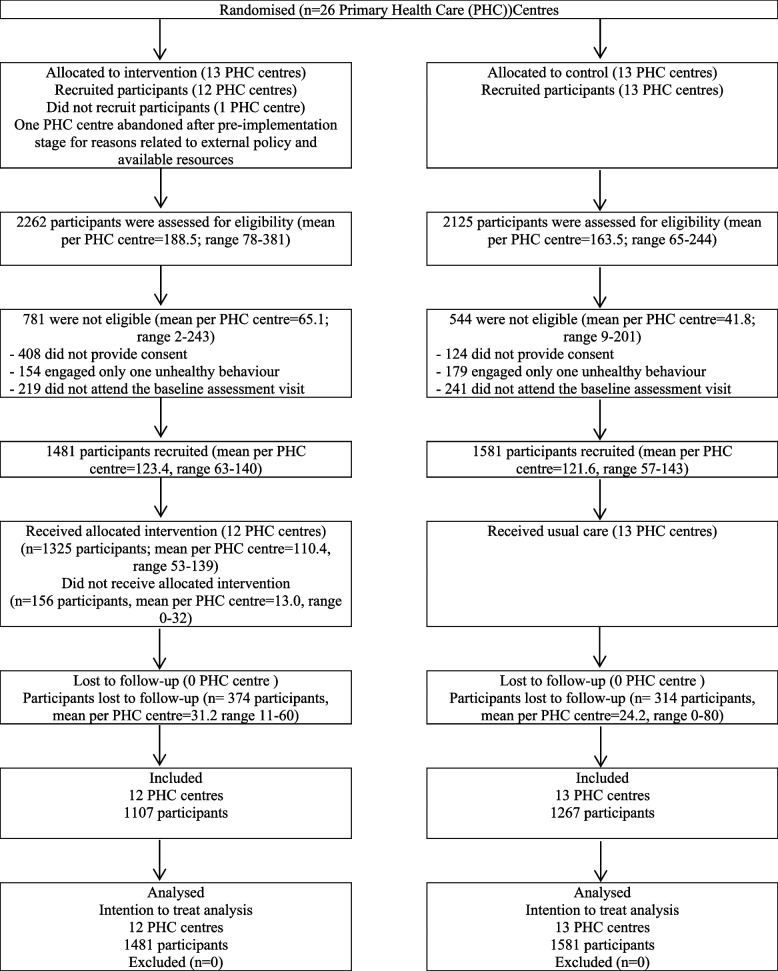
Flow diagram of clusters and participants through study

A total of 26 Spanish healthcare centres participated (13 IG and 13 CG). One centre of the IG could not recruit any participants for reasons related to external policy and available resources. A total of 4,387 participants were evaluated for eligibility, of whom 532 did not give their consent, 333 had only one unhealthy behaviour and 460 did not attend the baseline assessment visit. Finally, 3,062 participants were included (1,481 in IG and 1,581 in CG) (Fig. 1).

Table 2 shows the characteristics of the population. The mean age of the participants was 58.0 ± 8.1 years (54.9% women) without differences between the study groups. Moreover, there were no differences in marital status, with a predominance of married/coexisting participants (68.5%), work situation (48.0% employed), or educational level (39.4% with secondary or higher education). However, as is shown in Table 2, there were more IG than CG participants in the stage of preparation for changing dietary habits.

**Table 2:** Baseline characteristics of the study population

	**EIRA Intervention (*n* = 1481)**	**Usual care (*n* = 1581)**
Age (years)	57.7 (7.9)	58.3 (8.3)
Females (n, %)	809 (54.6%)	872 (55.2%)
Body mass index (Kg/m^2^)	30.6 (5.9)	29.3 (6.0)
Diabetes (n, %)	277 (18.7%)	323 (20.4%)
Hypertension (n, %)	587 (39.6%)	610 (38.6%)
Marital status (n, %)		
Single	139 (9.4%)	202 (12.8%)
Married	1024 (69.1%)	1055 (66.6%)
Separated or divorced	211 (14.3%)	202 (12.8%)
Widower	86 (5.8%)	115 (7.3%)
Other	1 (0.1%)	1 (0.1%)
No answer	20 (1.4%)	6 (0.4%)
Work situation (n, %)		
Employed	661 (44.6%)	713 (45.1%)
Unemployed	145 (9.8%)	141 (8.9%)
Homemaker	174 (11.7%)	194 (12.2%)
Retired	371 (25.1%)	431 (27.3%)
Other	105 (7.1%)	96 (6.1%)
No answer	25 (1.7%)	6 (0.4%)
Educational level completed (n, %)		
University studies	247 (16.7%)	267 (16.9%)
Middle or High school	573 (38.6%)	621 (39.3%)
Elementary school	547 (36.9%)	599 (37.8%)
None	94 (6.4%)	85 (5.4%)
No answer	20 (1.4%)	9 (0.6%)
Dietary habits stage of change (n, %)^a^		
Precontemplation	98 (6.6%)	554 (35.1%)
Contemplation	220 (14.9%)	295 (18.7%)
Preparation	619 (41.8%)	206 (13.0%)
Action	244 (16.5%)	179 (11.3%)
Maintenance/termination	89 (6.0%)	318 (20.1%)
No answer	211 (14.2%)	29 (1.8%)

Categorical variables are expressed as n (%) and continuous variables as mean ± standard deviation

^a^Statistically significant differences (*p* < 0.05)

### Description of the components of the intervention

Among the 1,481 IG participants, 843 (56.9%) showed co-occurrence of non-adherence to the MD and insufficient physical activity, 136 (9.2%) non-adherence to the MD and smoking and 405 (27.3%) exhibited all 3 behaviors at the same time. Low adherence to the MD was present in 1,384 (93.5%) participants, of whom 1,233 initiated the intervention and conducted at least one individual visit with a healthcare professional of the healthcare centre, in which aspects related to the MD were addressed. More than half of the participants received written material related to the intervention, 313 participants were referred a group session about lifestyles and 113 were referred to community programmes. Among the recommendations for the use of new technologies, 40.5% agreed to receive text messages and only 25.6% received recommendations for the download and use of smartphone applications (Table 3).

**Table 3:** Description of the EIRA Intervention

	**EIRA Intervention (*n* = 1481)**
Co-occurrence of unhealthy behaviours	
Non-adherence to Mediterranean dietary pattern & insufficient physical activity	843 (56.9%)
Non-adherence to Mediterranean dietary pattern & smoking	136 (9.2%)
Smoking & insufficient physical activity	97 (6.5%)
Non-adherence to Mediterranean dietary pattern & insufficient physical activity & smoking	405 (27.3%)
Agree to receive text messages on the mobile phone	600 (40.5%)
Recommendation to enter the study’s web platform	415 (28.0%)
Frequently accessed the study’s web platform	68 (4.6%)
Recommendation to use smartphone APPs	380 (25.7%)
Use smartphone APPs	20 (1.36%)
Non-adherent Mediterranean dietary pattern	1384 (93.5%)
Intervention started	1233 (89.1%)
Referral to community programs	113 (8.2%)
Referral to group session	313 (22.6%)
Attend the group session	44 (3.0%)
Written material delivered	751 (54.3%)

### Adherence to the MD in the baseline evaluation

No differences were detected in the total score of the questionnaire of adherence to the MD at the baseline evaluation between the study groups, with IG and CG obtaining 6.74 and 6.79 points, respectively. Differences were found in the number of participants who obtained at least 9 points in the questionnaire (IG = 240 (16.2%) vs. CG = 316 (20.0%), (*p* < 0.05)). Among the 14 recommendations that establish good adherence to the MD, the use of virgin olive oil as the main fat to cook and season foods reached the highest percentage of compliance, with 91.4% in both groups. On the other hand, the items that showed the lowest scores were those related to the weekly consumption of legumes and wine. Three of the fourteen items showed differences in compliance between the study groups, with IG obtaining the highest compliance percentage regarding the intake of olive oil and CG obtaining the highest compliance percentage regarding the daily consumption of carbonated and/or sugary beverages (Table 4).

**Table 4:** Compliance with each item of the Mediterranean diet adherence screener

Outcome measure	EIRA Intervention (*n* = 1481)		Usual care (*n* = 1581)	
	Baseline	12 months	Baseline	12 months
Study participants with a total score ≥ 9 points, n (%)*	240 (16.2%)	628 (42.4%)	316 (20.0%)	543 (34.4%)
Use of olive oil as the main fat to cook, n (%)	1354 (91.4%)	1257 (84.9%)	1445 (91.4%)	1376 (87.0%)
Daily consumption of at least 4 spoonfuls of olive oil, n (%) *	859 (58.0%)	901 (60.8%)	848 (53.6%)	878 (55.5%)
Daily intake of at least 2 servings of vegetables (at least one of them in a salad or raw), n (%)	395 (26.7%)	573 (38.7%)	410 (25.9%)	558 (35.3%)
Daily consumption of 3 or more fruit pieces (including natural juice), n (%)	367 (24.8%)	625 (42.2%)	398 (25.2%)	610 (38.6%)
Consumption of less than one ration of red meat, hamburgers, sausages or processed meat per day, n (%)	947 (63.9%)	1171 (79.1%)	1018 (64.4%)	1237 (78.2%)
Consumption of less than one ration of cream, butter or margarine per day, n (%) *	1181 (79.7%)	1311 (88.5%)	1333 (84.3%)	1412 (89.3%)
Intake of less than one carbonated and/or sugary beverage per day, n (%) *	1018 (68.7%)	1185 (80.0%)	1199 (75.8%)	1293 (81.8%)
Consumption of 7 or more glasses of wine per week, n (%)	287 (19.4%)	359 (24.2%)	324 (20.5%)	382 (24.2%)
Weekly consumption of at least 3 servings of legumes, n (%)	321 (21.7%)	469 (31.7%)	316 (20.0%)	397 (25.1%)
Consumption of at least 3 servings of fish or shellfish per week, n (%)	535 (36.1%)	696 (47.0%)	548 (34.7%)	648 (41.0%)
Intake of industrial (non-home-made) pastries less than twice per week, n (%)	705 (47.6%)	974 (65.8%)	770 (48.7%)	1002 (63.4%)
Intake of nuts 3 or more times per week, n (%)	373 (25.2%)	567 (38.3%)	420 (26.6%)	487 (30.8%)
Preference for the consumption of chicken, turkey or rabbit meat instead of beef, pork, hamburgers or sausages, n (%)	883 (59.6%)	1112 (75.1%)	924 (58.4%)	1154 (73.0%)
Consumption of “sofrito” (sauce made with tomato, garlic, onion and other vegetables to dress rice, pasta, meat and other dishes) 2 or more times per week, n (%)	756 (51.1%)	808 (54.6%)	786 (49.7%)	817 (51.7%)
**Score for adherence to Mediterranean Diet**^a^	6.74 (0.51)	8.11 (0.69)	6.79 (0.50)	7.75 (0.61)

^a^Expressed as mean (standard error)

^*^p < 0.05 at baseline evaluation between groups

### Effectiveness of the intervention regarding changes in adherence to the MD

A greater increase (13.7%; 95% CI, 9.9 to 17.5; *p* < 0.001) was obtained by IG in the number of participants who reached 9 points or more in MEDAS at 12 months. Moreover, the effect attributable to the intervention, after controlling for age, sex, centre and dietary habits stage of change obtained a greater increase (0.50 points; 95% CI, 0.35 to 0.66; *p* < 0.001) in IG (Table 5). The most relevant effects were observed in the items related to the weekly consumption of nuts, with IG obtaining a greater increase (9.4%; 95% CI, 5.9 to 12.9; *p* < 0.001) in the number of participants who complied with this recommendation. IG also showed an increase in the items related to the daily consumption of fruit, red and processed meat, the intake of butter, margarine and/or cream, the consumption of carbonated and/or sugary drinks, the weekly consumption of legumes and the weekly consumption of fish or shellfish and pastries.

**Table 5:** Intervention attributable difference in good adherence and compliance with each item

	**Intervention attributable difference (IG-CG) (% (95% CI))**	***p* value**	**Intervention attributable difference (IG-CG) (% (95% CI))** **Adjusted for age, sex, centre and dietary habits stage motivation**	***p* value**
**Outcome measure**				
Study participants with a total score ≥ 9 points (%)	11.9 (7.7 to 16.0)	< 0.001	13.7 (9.9 to 17.5)	< 0.001
Use of olive oil as the main fat to cook, n (%)	-2.2 (-5.7 to 1.3)	0.222	-0.2 (-0.5 to 0.0)	0.080
Daily consumption of at least 4 spoonfuls of olive oil, n (%)	1.0 (-3.3 to 5.2)	0.660	-0.2 (-4.1 to 3.6)	0.902
Daily intake of at least 2 servings of vegetables (at least one of them in a salad or raw), n (%)	2.7 (-1.7 to 7.0)	0.230	2.8 (-1.0 to 6.5)	0.148
Daily consumption of 3 or more fruit pieces (including natural juice), n (%)	4.1 (-0.1 to 8.3)	0.058	4.7 (1.0 to 8.4)	0.014
Consumption of less than one ration of red meat, hamburgers, sausages or processed meat per day, n (%)	1.3 (-2.8 to 5.4)	0.524	4.0 (0.4 to 7.5)	0.027
Consumption of less than one ration of cream, butter or margarine per day, n (%)	3.8 (0.4 to 7.2)	0.027	4.2 (1.3 to 7.1)	0.005
Intake of less than one carbonated and/or sugary beverage per day, n (%)	5.3 (1.8 to 8.9)	0.003	5.9 (2.7 to 9.0)	< 0.001
Consumption of 7 or more glasses of wine per week, n (%)	1.2 (-2.6 to 5.0)	0.537	1.4 (-1.5 to 4.2)	0.350
Weekly consumption of at least 3 servings of legumes, n (%)	4.9 (0.7 to 9.0)	0.022	5.7 (2.3 to 9.1)	< 0.001
Consumption of at least 3 servings of fish or shellfish per week, n (%)	4.5 (0.3 to 8.7)	0.038	5.3 (1.6 to 8.9)	0.005
Intake of industrial (non-home-made) pastries less than twice per week, n (%)	3.5 (-0.8 to 7.8)	0.113	6.1 (2.2 to 10.1)	0.002
Intake of nuts 3 or more times per week, n (%)	8.9 (5.0 to 12.8)	< 0.001	9.4 (5.9 to 12.9)	< 0.001
Preference for the consumption of chicken, turkey or rabbit meat instead of beef, pork, hamburgers or sausages, n (%)	0.9 (-3.1 to 5.0)	0.647	3.0 (-0.7 to 6.7)	0.112
Consumption of “sofrito” (sauce made with tomato, garlic, onion and other vegetables to dress rice, pasta, meat and other dishes) 2 or more times per week, n (%)	1.5 (-3.0 to 6.1)	0.510	2.2 (-1.8 to 6.1)	0.278
**Score for adherence to Mediterranean Diet**	0.41 (0.24 to 0.59)	< 0.001	0.50 (0.35 to 0.66)	< 0.001

### Effectiveness of the intervention on secondary variables (laboratory and weight variables)

After applying an analysis model controlled for age, sex, centre, stage of change in dietary habits, and change in physical activity and smoking cessation, we observed changes attributable to the intervention in one of the secondary variables studied, which are related to cardiovascular risk factors. There was a greater decrease in systolic blood pressure (-2.4 mmHg; CI 95%, -4.3 to -0.5; *p* = 0.014). No relevant effects were observed in the lipid profile, weight or the glycated haemoglobin (Table 6).

**Table 6:** Change in secondary outcomes (laboratory variables and weight) from baseline to 12-month follow-up

							**Intervention attributable difference Mean (95% CI)**	***p* value**	**Intervention attributable difference (IG-CG) (% (95% CI))** **Adjusted for age, sex, centre, dietary habits stage motivation and change in physical activity and smoking cessation**	***p* value**
**Outcome measure**	**EIRA Intervention (*n* = 1481)**			**Usual care (*n* = 1581)**						
	Baseline	After 12 months	Difference	Baseline	After 12 months	Difference				
**Total cholesterol (mg/dL)**	206.6 (3.8)	203.7 (20.0)	-2.9 (20.8)	206.5 (4.2)	205.8 (20.0)	-0.7 (21.2)	-2.2 (-10.4 to 6.1)	0.603	-1.2 (-4.6 to -2.3)	0.515
**LDL-cholesterol (mg/dL)**	127.3 (4.8)	123.1 (21.4)	-4.2 (23.0)	127.2 (4.9)	125.6 (20.7)	-1.6 (22.2)	-2.6 (-10.8 to 5.6)	0.531	-1.7 (-5.7 to 2.3)	0.395
**HDL-cholesterol (mg/dL)**	53.0 (1.1)	54.0 (5.4)	1.0 (5.7)	53.7 (1.4)	54.6 (5.3)	0.9 (5.5)	0.0 (-2.8 to 2.8)	0.990	0.1 (-1.1 to 1.3)	0.905
**Triglycerides (mg/dL)**	148.2 (17.2)	187.7 (49.3)	39.5 (49.4)	146.5 (19.4)	178.9 (47.2)	32.4 (48.5)	7.1 (-17.2 to 31.4)	0.563	5.7 (-7.0 to 18.3)	0.380
**Glycated haemoglobin (%)**	7.5 (0.4)	7.4 (0.8)	-0.1 (0.8)	7.5 (0.4)	7.4 (0.8)	-0.0 (0.8)	-0.1 (-0.6 to 0.4)	0.771	-0.1 (-0.4 to 0.3)	0.676
**Systolic blood pressure (mmHg)**	133.5 (1.4)	131.6 (6.9)	-1.9 (7.3)	131.9 (0.7)	132.1 (5.7)	0.2 (5.8)	-2.1 (-6.0 to 1.8)	0.286	-2.4 (-4.3 to -0.5)	0.014
**Diastolic blood pressure (mmHg)**	82.5 (0.7)	81.9 (3.9)	-0.6 (3.9)	80.5 (0.4)	80.9 (3.1)	0.4 (3.1)	-1.0 (-3.3 to 1.4)	0.414	-1.2 (-2.6 to 0.1)	0.073
**Weight (kg)**	82.3 (0.6)	82.1 (5.9)	-0.2 (6.0)	78.8 (0.4)	79.1 (4.8)	0.3 (4.8)	-0.5 (-4.0 to 2.9)	0.764	-0.4 (-2.5 to 1.7)	0.711

Data expressed as mean (standard error)

## Discussion

The main finding of this study is the improvement of adherence to the MD caused by a complex intervention which was conducted within a real clinical context of primary health care by professionals of Spanish healthcare centres. The intervention, which included an individual, group and community approach, was also able to modify other clinical parameters related to the effects of cardiovascular risk factors, like systolic blood pressure.

The MD has become one of the most studied healthy dietary patterns and a target within the improvement of dietary behavior, mainly due to its cardiovascular effects [41, 42]. However, there are few studies focused on improving the adherence to this dietary pattern. The existing studies include very different populations and types of intervention [43–46]. In this sense, research has been focused on the study of populations at high cardiovascular risk, such as overweight or obese people [47] or people with type 2 diabetes mellitus [6], and thus very few interventions include general adult populations. With respect to the type of intervention, some of these interventions use information and communication technologies, such as websites for the participants, text messaging, smartphone applications [7], and others include additional dietary supplements in the habitual diet with primary components of MD, such as nuts or olive oil [48]. However, these interventions are difficult to implement in usual clinical practice due to their cost. Complex interventions, like that of the EIRA study, are aimed at solving some of these difficulties from their design. In the EIRA study, the intervention was modelled based on the characteristics of the population attended in each healthcare centre, with common elements for all. Another fundamental aspect to understand the nature of complex interventions is that they are performed within a real clinical healthcare context so that the EIRA intervention was part of the daily healthcare job of physicians, nurses, social workers and other professionals like the administration staff of the participating primary healthcare centres, which is an advantage for the implementation of its results.

In terms of public health, the effect attributable to the intervention in terms of increasing adherence to the MD is not very relevant (0.50 points). The meta-analysis by Sofi et al. [2] concluded that a 2-point increase in the MD adherence score was associated with a 10% reduction in cardiovascular disease incidence and mortality, so such a small half-point increase could have a very minor impact. However, very few studies have found relevant differences. The MedLey study, whose intervention consisted of a dietitian-led intervention where all participants consulted the study dietitian fortnightly, received resources including recipes and meal plans, and those in the intervention arm collected study foods representing the MD, achieved an increase of 1.3 points in the adherence questionnaire [43]. While the reseach of Lugones-Sanchez et al. [45] and Gonzalez-Ramirez et al. [44], with interventions based on the use of smartphone applications, achieved very slight increases of 0.37 and 0.40 points, respectively, in the experimental group. The most important results were achieved by the study of Chou et al. [46], which achieved an increase of 2.22 points with an intervention based on game-based learning strategies that included motivation promotion, interactive self-learning, guiding questions and reflection between educators and learners about the progress of the game activity.

However, the effect on the number of people who, after the EIRA intervention, obtained good adherence to the MD (13.7%) is of greater importance. Among the 14 points of which the MEDAS is constructed, significant increases in compliance were achieved in 13. However, in the first one, regarding the use of olive oil as the main fat for cooking, this was not the case. A possible explanation for this finding is that it is the item which already had the highest compliance at baseline (91.4%). It is difficult to know what specific aspects of the intervention may condition these results, but a possible explanation for this finding is that during the individual intervention visit, personalized objectives were established that included the modification of no more than 2 or 3 of the 14 elements present in the MD adherence questionnaire. These objectives were aimed at meeting those aspects of adherence to the MD that were not met at the baseline visit. It is necessary to discuss the influence that some socioeconomic factors may have had on the change in adherence to the MD, especially in Mediterranean populations like that of the EIRA study. There are several papers that have previously analyzed this in greater detail. Absolute change in the MD score was positively associated with female gender, age, higher education, and moderate physical activity [49–51]. On the other hand, there is evidence that the recruitment of individuals with high perceived self-efficacy to dietary change, and those who initially follow diets relatively richer in fiber may lead to greater changes in nutritional recommendations [52].

A slight reduction (-2.4 mmHg (CI -4.3 to -0.5)) in systolic blood pressure figures favorable to the IG has been observed. These results are similar to the findings of other recent studies, such as the MedLey study [43]. However, and despite including an adjustment for positive changes in physical activity and smoking cessation in the analysis model, we cannot firmly state that this reduction in systolic blood pressure is a consequence of the change in diet.

Among the main limitations of this study we find the following. In reference to the selection of participating centres, those responsible for the study selected the centres based on a series of circumstances that included areas in which there was not a great cultural and linguistic diversity, and this may represent a possible selection bias that influences the generalization of the results. Second, the assessment of adherence to the MD was carried out through a questionnaire, which, although validated for the reference population, does not guarantee correct adherence as accurately as other nutrient biomarkers can. This study recruited low fruit and vegetable consumers who can still score 9 points or more on MEDAS (i.e., have high adherence to the MD). This may also constitute a possible selection bias. The intervention could have benefited from the direct participation of nutritionists or dieticians in the direct transfer of knowledge of the Mediterranean Diet to the participants, although the professionals involved were trained by a dietitian.

## Conclusions

A complex intervention, designed, modelled and carried out by primary healthcare professionals, within a real clinical healthcare context, showed an increase in the percentage of participants that achieve good adherence to the MD compared to the brief advice given in the usual visits to the doctor’s office, although the overall change in the MEDAS was small.

## Data Availability

The datasets used and analysed during this study are available from the corresponding author on reasonable request.
